# Exploring primary care physicians’ challenges in using home blood pressure monitoring to manage hypertension in Singapore: a qualitative study

**DOI:** 10.3389/fmed.2024.1343387

**Published:** 2024-03-25

**Authors:** Aminath Shiwaza Moosa, Prawira Oka, Chirk Jenn Ng

**Affiliations:** ^1^SingHealth Polyclinics, Singapore, Singapore; ^2^SingHealth Duke-NUS Family Medicine Academic Clinical Programme, Singapore, Singapore

**Keywords:** home blood pressure, hypertension management, primary care, family medicine, self-monitored, uncontrolled hypertension

## Abstract

**Objective:**

Hypertension guidelines recommend using home blood pressure (HBP) to diagnose, treat and monitor hypertension. This study aimed to explore the challenges primary care physicians (PCPs) face in using HBP to manage patients with hypertension.

**Method:**

A qualitative study was conducted in 2022 at five primary care clinics in Singapore. An experienced qualitative researcher conducted individual in-depth interviews with 17 PCPs using a semi-structured interview guide. PCPs were purposively recruited based on their clinical roles and seniority until data saturation. The interviews were audio-recorded, transcribed verbatim and managed using NVivo qualitative data management software. Analysis was performed using thematic analysis.

**Results:**

PCPs identified variations in patients’ HBP monitoring practices and inconsistencies in recording them. Access to HBP records relied on patients bringing their records to the clinic visit. A lack of seamless transfer of HBP records to the EMR resulted in an inconsistency in documentation and additional workload for PCPs. PCPs struggled to interpret the HBP readings, especially when there were BP fluctuations; this made treatment decisions difficult.

**Conclusion:**

Despite strong recommendations to use HBP to inform hypertension management, PCPs still faced challenges accessing and interpreting HBP readings; this makes clinical decision-making difficult. Future research should explore effective ways to enhance patient self-efficacy in HBP monitoring and support healthcare providers in documenting and interpreting HBP.

## Introduction

Treatment of hypertension relies heavily on accurate BP measurement. However, office BP alone is inadequate to assess BP control accurately due to BP variability and the existence of various hypertension phenotypes such as whitecoat uncontrolled hypertension and masked hypertension (1–3). Increasingly, out-of-office BP measurements, including ambulatory BP and home BP (HBP), are being recommended in the diagnosis and monitoring of hypertension (4). Additionally, high ambulatory BP and HBP have been associated with increased cardiovascular morbidity and mortality independent of office BP measurements (5, 6). HBP monitoring is superior to ambulatory BP monitoring in cost, accessibility and usability (7, 8). Globally, HBP device ownership varies between 30 and 70% (9–15). As a population known for embracing technology in a local study, more than 80% of Singaporean physicians recommended HBP monitoring to their patients and utilized these measurements to monitor the effects of anti-hypertensive therapy and make informed clinical decisions (16).

However, despite the increasing reliance on using HBP to diagnose, monitor and treat hypertension, studies have reported that PCPs face challenges in using HBP to manage patients with hypertension (17, 18). These included inconsistency in HBP measurement, high out-of-pocket costs of home BP devices, and time needed to instruct patients on home BP monitoring procedures. A more recent study among American PCPs reported difficulty accessing patients’ HBP records and a lack of workflow to support patients using HBP devices (19). A local cross-sectional survey of 60 physicians (30 PCPs, 20 cardiologists and 10 nephrologists) identified patient inertia, poor patient adherence, short medical consultation time, and poor patient access to a BP machine as the barriers to implementing HBP monitoring in both hospital and primary care settings (16).

In primary care, where the majority of patients with hypertension are managed, the importance of HBP to inform prompt and accurate clinical decisions is even more pertinent, given the time constraints. Therefore, this study aimed to explore the challenges faced by PCPs when using HBP to manage patients with hypertension in their daily clinical practice. The findings of this study will help in the design of effective interventions in supporting healthcare providers to make informed decisions based on HBP. This study is part of a larger study to explore the challenges faced by doctors, nurses and pharmacists in managing patients with hypertension in a primary care setting.

## Materials and methods

### Study design

A qualitative methodology using the descriptive-interpretive approach (20) explored the challenges in managing patients with hypertension, particularly on HBP.

### Study site

Individual in-depth interviews (IDIs) of PCPs were conducted across five public primary care clinics located in the south and eastern region of Singapore (Bukit Merah, Marine Parade, Eunos, Sengkang and Pasir Ris). These primary care clinics provided hypertension care for a multi-ethnic Asian population; in 2022, there were over 200,000 patient-visits for hypertension with 850 attendances daily (based on the institution’s electronic medical record (EMR) system and business database).

### Period of study

The field work was conducted between April 2022 and March 2023.

### Research team

The research team comprised one female (ASM) and two male (PO, CJN) PCPs who worked in the institution where the study was conducted; they managed patients with hypertension in their routine clinical practice. All researchers are trained in qualitative research.

### Study population and recruitment

The target study population was PCPs actively practicing in an urban public primary care setting. The eligible participants were PCPs aged 21 years and older who had been actively involved in managing patients with hypertension for the past 6 months. An email invitation containing the study purpose were sent by the senior author (CJN) to the PCPs to solicit participant interest. They were informed that participation was voluntary. Those who expressed interest were approached by a research coordinator to schedule a face-to-face interview and obtain informed consent. PCPs were recruited purposively based on their seniority and roles with an attempt to achieve maximal variation; the recruitment continued until no new themes emerged from the field notes and analysis (data saturation).

### Study instruments

#### Participant demographic data collection form

Participant demographic data collection forms were used to collect information about PCPs’ age, gender, clinical experience, qualification and designation.

#### Topic guide

The researcher used a semi-structured topic guide to guide the IDIs. The topic guide was developed based on Theory of Planned Behavior (21), literature review and discussion with the team members. The questions included PCP’s experiences, barriers and facilitators, and their needs when managing patients with hypertension in general and specifically on HBP monitoring. The topic guide was pilot-tested and iteratively modified based on data that emerged during both pilot and subsequent interviews.

### Data collection

After obtaining the written consent, the participant completed the demographic data collection form, followed by the individual IDIs. The interviews were conducted face-to-face in a private room in the PCP’s practicing clinic. NCJ conducted all the interviews using the interview topic guide. Each interview lasted 45–60 min and all were audio-recorded. All participants were reimbursed with a grocery voucher of SGD20 (estimated USD15) to compensate for their time and effort.

### Data analysis

All interviews were audio-recorded, transcribed verbatim and checked for accuracy. Three researchers (ASM, OP, CJN) read and reread three transcripts independently and repeatedly to familiarize themselves with the data. Each researcher coded the transcript line-by-line to generate a list of initial codes (open coding). The research team met regularly to discuss the codes; any coding discrepancies were resolved via consensus. Codes with similar content were grouped into categories; these categories were further rearranged to create an Initial coding frame (axial coding). Based on the coding frame, the rest of the transcripts were divided and analysed individually by the three researchers (ASM, OP, CJN) using the NVivo© (QRS Pty Ltd., Australia) qualitative data management software. The researchers performed constant comparison of the content of each code, which were reviewed, revised, refined, and re-named through discussion and consensus. Finally, a report of the themes generated, including the challenges faced by PCPs when using HBP to manage patients, was written with representative quotes to illustrate the key findings. No new challenges emerged after 12 interviews; further five PCPs were interviewed to ensure the data has reached saturation (22, 23).

### Rigor

For the data collection, a senior researcher conducted all the interview; this helped to maintain the quality and consistency of the interviews. During the analysis, the research team met regularly to share, discuss and ‘challenge’ their data interpretations. The team adopted an open approach during the meetings and constantly reflected and debated potential biases they might carry due to their background as PCPs. The demographic data collection forms, audio recordings, transcripts, field notes, coding frame and codes were maintained in secure archives to establish a clear audit trail.

## Results

A total of 19 PCPs were approached, and 17 agreed and participated in the IDIs (response rate 89%). They were aged between 29 years and 80 years and had between 8 months and 40 years of clinical experience in primary care. The details of their demographic characteristics are presented in Table 1.

**Table 1:** Characteristics of participating primary care physicians (*n* = 17).

Characteristic	*n*	%
Gender		
Male	5	29%
Female	12	71%
Age (in years)		
Age < 35	8	47%
Age 35–49	7	41%
Age ≥ 50	2	12%
Median age	35	
Highest qualification		
Medicine of Bachelor and Medicine of Surgery (MBBS) or equivalent	3	18%
Graduate Diploma in Family Medicine (GDFM)^a^	4	24%
Master of Medicine in Family Medicine (MMed (FM))^b^	5	29%
Fellow of College of Family Physicians, Singapore (FCFPS)^c^	5	29%
Designation		
Medical Officer	1	6%
Resident Physician	2	12%
Family Physician (GDFM)	4	24%
Family Physician (MMed)	4	24%
Associate Consultant	3	18%
Consultant	3	18%
Years of practice (in years)		
< 10	13	76%
10–19	3	18%
≥ 20	1	6%
Median years of practice	7	

^a^Graduate Diploma in Family Medicine (GDFM) is a basic training program for primary care doctors in Singapore to improve their competency in Family Medicine.

^b^Master of Medicine in Family Medicine (MMed (FM)) is a Master level training for primary care doctors in Singapore to become experts in the field of Family Medicine.

^c^Fellow of College of Family Physicians, Singapore (FCFPS) is an advanced specialist level training for primary care doctors in Singapore to become leaders in the field of Family Medicine.

**Figure 1:**
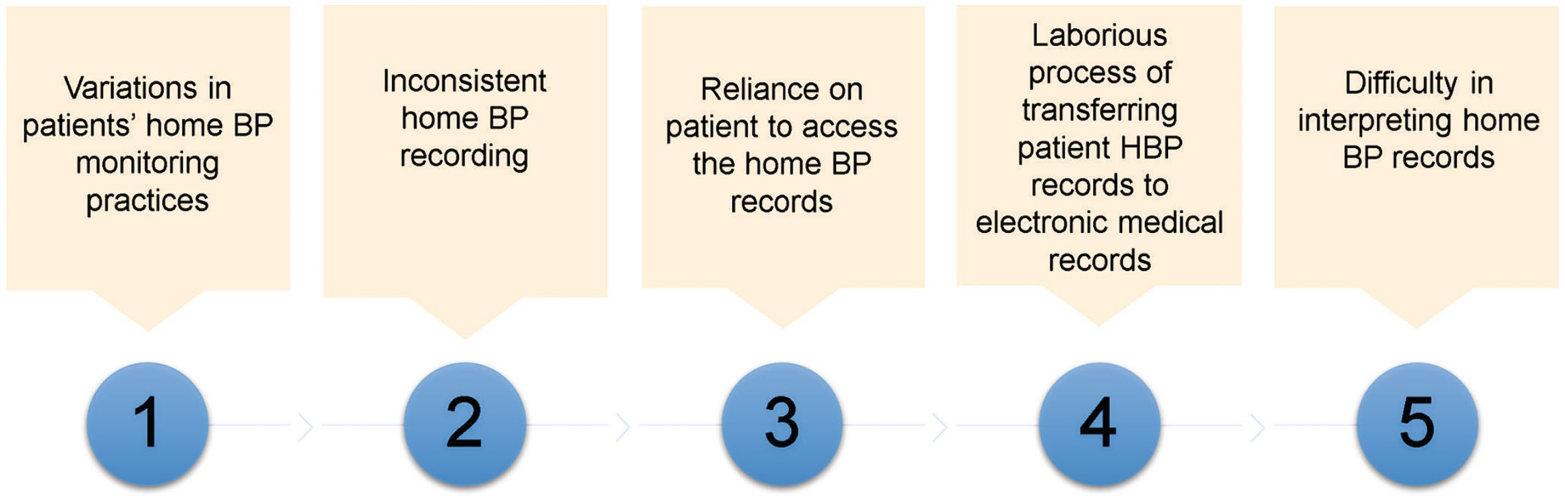
Challenges faced by primary care physicians on managing patients based on home blood pressure.

PCPs faced five main challenges when using HBP to manage patients with hypertension during their daily practice. They were: variations in patients’ home BP monitoring practices, inconsistent home BP recordings, reliance on patient to access the HBP records, laborious process of transferring patient HBP records to EMR, and difficulty in interpreting HBP records (Figure 1).

### Variations in patients’ HBP monitoring practices

PCPs reported a variation in patient adherence to HBP monitoring and how they measured BP. PCPs encountered patients who declined to monitor their HBP despite owning a BP set; they perceived this was due to a lack of patient motivation.

*“it’s very varied practice, some say they own a set but they do not use it because nobody told them to use it or they do not feel the need to use it or they use it occasionally when they do not feel well.,”* P08 (47-year-old, Consultant, 17 years of practice).

*“Some of them (patients) will just dismiss and say, “I really do not have the time, I’m too tired.” For these patients, I feel that they do not really have the motivation...”* P07 (36-year-old, Associate Consultant, 11 years of practice).

*“I was told that he has a machine, but just too lazy, not motivated to check. So, this time he admitted, “Actually, I’m not even (checking), do not know where the machine is actually.”* P17 (32-year-old, Family Physician, 4 years of practice).

PCPs were also concerned that patients might not be measuring the HBP correctly or the HBP monitoring devices might not be calibrated; this made them doubt the accuracy of the HBP readings.

*“… because a lot of times if they do it wrongly or the machine is faulty then of course you are going to get very erroneous numbers. Sometimes, the artery marker is placed on the other side that is one thing. Sometimes it could be the position of their arm whether it’s at the heart level or not.”* P14 (35-year-old, Family Physician, 8 years of practice).

### Inconsistency in HBP documentation by patients

PCPs also reported a wide variation in how patients document their HBP readings. PCPs struggled to make treatment decisions when patients did not document their BP or bring their BP records during their clinic visit.

*“And they may or may not, in fact more of them do not record their blood pressure. So, that is a challenge because when they come in and their blood pressure is high and you ask them, “Oh, what is your blood pressure?.” Some will say, “Oh, at home it’s normal” and you ask them, “What is that number?” and they say, “I do not know but it’s normal.” So, how do you know it’s normal when you do not have a number, right?,”* P08 (47-year-old, Consultant, 17 years of practice).

*“A lot of times they thought that it’s (recording and bringing BP records) a hassle. Some will feel like it’s not going to change anything. “Anyway, I am only here to collect medicine,” but they do not understand the point that the BP reading, they are actually so valuable because they (make) me decide what to do with their medication.,”* P11 (34-year-old, Family Physician, 3 years of practice).

### Reliance on patient to access the HBP records

PCPs relied on their patients to bring their HBP records to the clinic. Despite the availability of institution-based digital HBP diaries (Health Buddy), the adoption of these digital tools was low especially among the older, less tech-savvy patient populations.

*“In general, we got a fairly healthy home BP ownership rate. But the problem lies more with patients forgetting to bring the home BP readings. “,* P16 (40-year-old, Consultant, 12 years of practice).

*“… I would say the take up rate or the use of these digital blood pressure diaries is still very low. Our population, most patients with hypertension are probably in the middle to elderly age group. Many of them are not that tech-savvy.”* P08 (47-year-old, Consultant, 17 years of practice).

### Laborious process of transferring patient HBP records to electronic medical record (EMR)

Currently, PCPs had to manually key in patients HBP records into the EMR during the consultation. They had limited time to review stacks of HBP records brought by patients and to document them in the EMR. Without proper documentation, PCPs would not be able to assess patients’ “BP trend” over time.

*“Even for the digital BP platform itself, the information goes to a separate platform but it’s not linked to the EMR, which means you need to open up another system. That is an inconvenience for the doctors,”* P16 (40-year-old, Consultant, 12 years of practice).

*“Sometimes my patient’s hypertension is not very well-controlled. Then, I will need to flip back (his old BP records). Some of them, bring the whole stack, then I will flip back the past few months to see where it went wrong. But some patients will just throw (the past BP records) away. There is no good way to store this information which the patient has spent so much effort providing you with,”* P07 (36-year-oild, Associate Consultant, 11 years of practice).

### Difficulty in interpreting home blood pressure

Some PCPs struggled to interpret the HBP readings, particularly when the HBP readings fluctuated significantly, or were at the borderline. PCPs were hesitant to use these BP measurements to make treatment decisions.

*“And then, they bring along a set of readings that looks high, sometimes low, sometimes you just do not know what to do.”* P07 (36-year-oild, Associate Consultant, 11 years of practice).

*“…I mean for those HBP readings that were a bit borderline, you cannot say with confidence whether they have hypertension or not. I mean they are some which are clear-cut but those like borderline, you cannot say for sure. I do not like to just put a label on the person if the evidence is not very strong.”* P15 (36-year-old, Family Physician, 6 years of practice).

There was a wide practice variation in how PCPs interpret the HBP readings; most would interpret the HBP by ‘eyeballing’ the BP readings. PCPs recognized this practice gap and attributed it to the lack of guidelines on HBP monitoring and interpretation.

*“And I would circle every reading that is above the clinical target. And after I’ve done that, I will look at the sheet of paper and ask myself, ‘Do I see more circled readings or non-circled readings?’ This is a very crude, rough way of doing it. Of course, the best way is to actually get the average but what is the practical way to do this very quickly in a 10-min consultation,”* P08 (47 year-old, Consultant, 17 years of practice).

*“Ya. I guess okay looking at the home readings… it’s like I have my own internal thing, I guess. If maybe less than 20% of the readings are too high then I’ll consider it as okay overall.’* P12 (37-year-old, Consultant, 9 years or practice).

*“Sometimes they will come with a paper. And then I would like review the readings to see the rough range of the readings, whether it is morning or at night and whether there is a dip in the nocturnal blood pressure or not. And then look at the majority average of the readings and see whether they are optimal or suboptimal. I do not think we have a guide on how it should be reviewed or assessed. So it might be doctor-specific.”* P05 (29-year-old, Family Physician, 3 years of practice).

## Discussion

Our study highlights practical challenges PCPs encountered when using HBP to manage patients with hypertension. While most of the identified challenges related to patient behavior, including variations in HBP measurement and documentation, the study also surfaced important gaps in PCP competency and a system in transferring and integrating HBP data. The PCPs in this study did not perceive cost and access to HBP monitoring devices as major challenges in using HBP to manage patients with hypertension.

Similar to our study, inconsistencies in HBP measurement and recording pose a barrier to using these records to manage patients with hypertension (24, 25). In a survey among 643 hypertensive patients, more than two-thirds (71%) reported incorrect home BP measuring techniques (18). In a qualitative study, PCPs believed that patients failed to follow correct protocols for rest and body positioning during HBP measurements (26). A local study by Setia et al. (16) found that PCPs perceived patient inertia and poor patient compliance as the most common barriers to implementing out-of-office BP monitoring. In addition, Al-rousan et al. (27) reported patients’ willingness to monitor HBP but had concerns about their ability to do so. While patient education by clinicians and nurses can improve HBP technique (25, 28), the time needed to instruct patients on HBP measuring protocols may not be feasible or available in busy clinical settings (18). Remote patient education through videos and online platforms could help improve HBP practices and techniques while working within the existing time constraints (29).

Lack of integration of HBP records into the EMR limited PCPs’ access to patient’s records. A systematic review of 12 studies identified similar challenges, including manually transferring patient-reported HBP data from multiple sources and devices and comparing them with office BP (30). This process was also considered laborious in busy primary care clinics (30). While integrating HBP into clinical care is an important goal, achieving integration is complex (31, 32). Teo et al. (32) reported local PCPs’ and patients’ appreciation of the convenience of an integrated platform where HBP was automatically captured and transferred to a dashboard for PCPs to review and manage patients’ HBP promptly; patients were reassured they were being monitored by the care team and PCPs perceived their time was better utilized. Nevertheless, the participants highlighted the challenges with the usability of the equipment, management portal and data access (32). Thus, while integrating HBP records into the EMR can improve the workflow for PCPs and improve patient clinical outcomes, the perspectives of PCP and patients need to be incorporated into designing such a system to enhance its long-term adoption (33).

Similar to our study findings, studies by Teo et al., Setia et al., and Fletcher et al. also reported uncertainty among clinicians in interpreting HBP readings, particularly in patients with borderline BP and BP variability (16, 30, 32). Global hypertension guidelines recommend target HBP for therapy to the level below the threshold used to diagnose hypertension. However, a variation in these diagnostic thresholds results in variation in therapeutic HBP targets. While American College of Cardiology/American Heart Association 2017 guidelines specify a daytime (awake) average ≥ 130 mmHg systolic or ≥ 80 mmHg diastolic BP (4), European Society of Cardiology /European Society of Hypertension, International Society of Hypertension and National Institute for Health Care Excellence define hypertension as an average HBP of ≥135 mmHg systolic or ≥ 85 mmHg diastolic BP (24, 34, 35). Discrepancy also exists in HBP interpretation across the guidelines. These differences include the required number and timing of readings, averaging methods, and controversy over the omission of first readings (36, 37). The lack of updated local clinical guidelines and PCP awareness of existing global guidelines creates practice variations in hypertension management among local PCPs (16). Local updated practice guidelines and frequent refreshers can help standardize clinicians’ management practices and keep them abreast of the current best clinical evidence (38).

PCPs in our study attributed challenges in HBP interpretation to insufficient time to calculate the average BP, which can result in clinical inertia and suboptimum BP control (30). A lack of clinical decision-support system can also create difficulty in interpreting HBP data in a timely and accurate manner. Manually going through patients’ HBP records is time-consuming and may not be practical in a busy primary care setting (30). Clinical decision support integrated into the clinician workflow could facilitate data interpretation and prompt decision-making (39).

Unlike previous studies on HBP monitoring, the PCPs in this study did not perceive out-of-pocket cost and access to HBP monitors as major challenges (13, 14). This could be due to the Singapore government’s active promotion of using technology for health management (40). Additionally, various government and philanthropic programs actively support patients with chronic conditions like hypertension afford and utilize digital health tools. Locally, a philanthropic-sponsored program offers self-monitoring devices, including HBP devices, to “needy” patients at half the original price (41). Since its launch in 2020, more than 400 patients per year have benefited from it (41). Thus, introducing reimbursement and financial assistance enhance the use of HBP among patients, particularly those from low socio-economic stratum.

### Limitations

The study has several limitations. Firstly, PCPs in this study were from a single primary care cluster in Singapore. To improve the transferability of the study findings, primary care clinics serving a diverse patient population were included, and PCPs with differing clinical roles and levels of experience were recruited. Future studies may be conducted in the private primary care sector in Singapore; this would uncover challenges unique to the PCPs and patients in the private sector. Secondly, this study focused on the challenges faced by PCPs and did not capture the challenges patients face in performing HBP monitoring. We are currently embarking on a study to explore patients’ experiences and challenges in HBP monitoring in primary care setting.

## Conclusion

Despite international guidelines recommending HBP to inform the diagnosis, treatment and monitoring of hypertension, PCPs face significant challenges in accessing adequate and accurately measured HBP readings; they also struggle to document and interpret them consistently in their busy clinical practice. Future studies should identify effective ways to use HBP to support patient self-care and facilitate improved care for patients with hypertension by developing practical guidelines and clinical decision-support systems on HBP monitoring.

## Data availability statement

The raw data supporting the conclusions of this article will be made available by the authors, without undue reservation.

## Ethics statement

The studies involving humans were approved by SingHealth Centralised Institutional Review Board (CIRB No: 2022/2517). The studies were conducted in accordance with the local legislation and institutional requirements. The participants provided their written informed consent to participate in this study.

## Author contributions

AM: Conceptualization, Formal analysis, Investigation, Writing – original draft, Writing – review & editing, Data curation. PO: Conceptualization, Formal analysis, Investigation, Writing – review & editing, Data curation. CN: Conceptualization, Formal analysis, Funding acquisition, Investigation, Methodology, Supervision, Writing – review & editing, Data curation.
