# Supplementary material for: Exploring primary care physicians’ challenges in using home blood pressure monitoring to manage hypertension in Singapore: a qualitative study
Source: Front Med (Lausanne). 2024 Mar 25;11:1343387. doi: 10.3389/fmed.2024.1343387 (PMC10999538; doi:10.3389/fmed.2024.1343387)
Supplement: Supplementary file 2 [file Data_Sheet_2.docx]

EmPaTHy-Hypertension Project

Exploring the challenges in managing hypertension among primary care healthcare professionals in Singapore: A qualitative study

# Interview Guide

## **Preamble**

- Ice-breaking and introduction
- Explain the aims of the study and the research methods
- Allow time for the participants to read the participant information sheet if necessary
- Emphasise there is no right or wrong answer; it is the personal views and experiences that the study is seeking
- Reassure the participants that they do not have to answer the questions if they do not wish to do so. There is no need for them to provide any explanation
- Explain that the participant can withdraw from the interview anytime
- Highlight that the interview will be audio-recorded for the purpose of transcribing, which is necessary for data analysis
- Give the participants the opportunity to ask questions before obtaining consent
- Obtain written consent from all participants, before starting the interview or filling in the data collection form
- Ask ‘any questions’ and state ‘we are going to start the interview now’

## **Questions**

1. Can you tell me about the patient profile at your polyclinic?
   1. Age group, ethnicity, education level, disease severity, health-seeking behaviour
2. How do patients with hypertension first present in your clinic?
3. How do you diagnose hypertension?
   1. Probe: What threshold of blood pressure? Which guideline do you follow?
4. Do you use any clinical practice guideline to guide your hypertension management?
   1. If yes, which guideline and why
   2. What do you think about the guideline? Useful or not? Why?
5. What is your approach to treating hypertension?
   1. Probe: Non-pharmacological – diet, exercise (be specific)
   2. Probe: Medications – which category, order of starting, mono- or dual therapy, how and when to step up
6. What are the challenges you face when managing patients with hypertension?
   1. Probe: Patient factor: Non-adherence to treatment/lifestyle modifications, etc.
   2. Probe: Doctor factor: Knowledge and skills, keeping updated with latest evidence, etc.
   3. Disease factor: Diagnosis of different categories of hypertension (e.g. white-coat, masked, nocturnal, non-dipper, morning surge), etc.
   4. System factor: Use of electronic medical records for patients with hypertension, PTEC, interprofessional care delivery, etc.
7. Can you suggest ways to improve the current management of hypertension?
   1. Probe: Change in current hypertension care delivery? Training? Technology?
   2. Probe: Do you need any support to provide better care for your patients
8. Do you have anything else to share with me?

## **Ending**

- Thank the participant for their time and contribution
- Debrief the participants if necessary
- Give the vouchers to the participant and ask them to acknowledge receipt by signing the document
